# Managing tomato bacterial wilt through pathogen suppression and host resistance augmentation using microbial peptide

**DOI:** 10.3389/fmicb.2024.1494054

**Published:** 2024-12-11

**Authors:** Ishan Tiwari, Ali Asger Bhojiya, Devendra Jain, S. L. Kothari, Mohamed A. El-Sheikh, Shalini Porwal

**Affiliations:** ^1^Amity Institute of Microbial Technology, Amity University Uttar Pradesh, Noida, India; ^2^U. S. Ostwal P. G. College, Mohanlal Sukhadia University, Chittorgarh, India; ^3^Department of Molecular Biology and Biotechnology, Maharana Pratap University of Agriculture and Technology, Udaipur, India; ^4^Amity Institute of Biotechnology, Amity University Jaipur, Jaipur, India; ^5^Botany and Microbiology Department, College of Science, King Saud University, Riyadh, Saudi Arabia

**Keywords:** peptide, phytopathogen, tomato, agriculture, bacterial wilt

## Abstract

The increasing health and environmental risks associated with synthetic chemical pesticides necessitate the exploration of safer, sustainable alternatives for plant protection. This study investigates a novel biosynthesized antimicrobial peptide (AMP) from *Lactiplantibacillus argentoratensis* strain IT, identified as the amino acid chain PRKGSVAKDVLPDPVYNSKLVTRLINHLMIDGKRG, for its efficacy in controlling bacterial wilt (BW) disease in tomato (*Solanum lycopersicum*) caused by *Ralstonia solanacearum*. Our research demonstrates that foliar application of this AMP at a concentration of 200 ppm significantly reduces disease incidence by 49.3% and disease severity by 45.8%. Scanning electron microscopy revealed severe morphological disruptions in the bacterial cells upon exposure to the AMP. Additionally, the AMP enhanced host resistance by elevating defense enzyme activities, leading to notable improvements in plant morphology, including a 95.5% increase in plant length, a 20.1% increase in biomass, and a 96.69% increase in root length. This bifunctional AMP provides dual protection by exerting direct antimicrobial activity against the pathogen and eliciting plant defense mechanisms. These findings underscore the potential of this biologically sourced AMP as a natural agent for combating plant diseases and promoting growth in tomato crops. To the best of our knowledge, this is the first study to demonstrate the use of a foliar spray application of a biosynthesized microbial peptide as biocontrol agent against *R. solanacearum*. This interaction not only highlights its biocontrol efficacy but also its role in promoting the growth of *Solanum lycopersicum* thereby increasing overall agricultural yield.

## 1 Introduction

Bacterial wilt (BW), caused by *R. solanacearum*, is a major soil-borne plant disease, particularly affecting crops like tomatoes (*Solanum lycopersicum*), which are of great economic and nutritional importance globally (Afari-Sefa et al., 2011; Khaliq et al., 2020). In India, the annual tomato production nears 19 million tons, yet bacterial wilt continues to devastate crop yields, especially in southern regions where this pathogen thrives in diverse environments (Naresh et al., 2019). Yield losses due to BW can range from 0 to 90%, depending on cropping practices, pathogen strains, soil conditions, and climate (Akanmu et al., 2023; Khaliq et al., 2023). The pathogen’s genetic diversity, ability to persist in soil and water, and wide host range make management difficult (Mamphogoro et al., 2020; Lal et al., 2022; Holeva et al., 2024; Ibrahim et al., 2021).

Traditional control strategies like chemical pesticides, crop rotation, and field sanitation have shown limited success. The excessive use of synthetic pesticides has resulted in environmental contamination, resistance development, and reduced biodiversity (Pandey et al., 2003; Montesinos et al., 2021). Therefore, finding alternative, more sustainable control methods is a priority (Singh et al., 2017; Khaliq et al., 2023). Biological control using antagonistic microorganisms has emerged as a safer and more eco-friendly approach, with strains from the *Lactobacillus* genus showing promising results in managing bacterial wilt (Yadav et al., 2024; Brooks et al., 2024; Gengmao et al., 2014).

Despite these advancements, significant gaps remain in our understanding of how microbial products can be used to enhance plant defense mechanisms while directly controlling pathogens. Antimicrobial peptides (AMPs) are increasingly recognized for their dual role in plant protection—acting both as direct antimicrobial agents and as elicitors of plant defense responses (Chaudhary et al., 2012; Verma et al., 2023). The multitargeted approach of AMPs, which includes pathogen inactivation and stimulation of host defenses, represents a novel strategy for disease management (Wu et al., 2023). However, studies specifically examining the ability of AMPs to enhance host resistance against bacterial wilt through upregulation of defense-related enzyme are scarce (Zhang et al., 2021).

In recent years, lactic acid bacteria (LAB), particularly *L. argentoratensis*, have drawn attention for their production of bioactive peptides with antimicrobial properties (Martínez-Castro et al., 2018; Vanitha et al., 2009). AMPs from *L. argentoratensis* have been shown to be stable, low-toxicity, and protease-resistant, offering potential as biocontrol agents against a range of plant pathogens (Tiwari et al., 2024). However, their specific role in modulating plant defense mechanisms, particularly in the context of BW management, remains underexplored.

The objective of this study is to evaluate the efficacy of a microbial peptide (PRKGSVAKDVLPDPVYNSKLVTRLINHLMIDGKRGK) extracted from *Lactiplantibacillus argentoratensis* strain IT as a biocontrol agent against *Ralstonia solanacearum* in tomato plants. We aim to assess whether the peptide can: Provide protection against bacterial wilt when applied as a foliar spray, and induce the expression of defense- and stress-related enzymes, enhancing host resistance.

The study investigates the impact of foliar application of this AMP on plant disease severity and growth, comparing treated plants to control groups. Additionally, we explore the novel application of this microbial peptide in controlling bacterial wilt while promoting plant health, addressing gaps in existing research on the use of AMPs in plant disease management. The findings of this study have significant practical implications for sustainable agriculture, particularly for smallholder farmers who often face challenges in disease management with limited access to synthetic pesticides. By utilizing antimicrobial peptides (AMPs) derived from *Lactiplantibacillus spp.*, farmers can adopt a safer and environmentally friendly alternative to combat bacterial wilt in tomato plants. This strategy empowers smallholder farmers to improve crop yields and quality while minimizing health risks associated with chemical pesticide use, ultimately supporting food security and promoting sustainable farming practices.

## 2 Materials and methods

### 2.1 Microbes and culturing media

In this research study, *L. argentoratensis* strain IT, derived from goat milk, was maintained at −40°C in lactobacilli de Man Rogosa Sharpe (MRS) broth TM Media (India) and gene sequence was submitted to NCBI with assertion number (OQ054583.1). The bacterial pathogen *R. solanacearum* strain BI0001 was used. Bacterial strains were cultured on Nutrient Agar for 24 h at 28°C and harvested from the surface to prepare suspensions adjusted to 10^8^ CFU/ml. *R. solanacearum* strain BI0001 was obtained from the Division of Plant Pathology collection at the Indian Agricultural Research Institute in New Delhi, India.

### 2.2 Synthesis of peptides

The peptide (PRKGSVAKDVLPDPVYNSKLVTRLINHLMIDGKRG), derived from *Lactiplantibacillus argentoratensis* strain IT, was synthesized following previously established methods (Tiwari et al., 2024). For sufficient yield, the strain was cultured in MRS medium with 1% (v/v) inoculum, incubated at 30°C for 48 h, and then processed by centrifugation to collect the supernatant. Chilled methanol was added to precipitate the peptides, and following incubation at −20°C, the resulting pellet was washed, dissolved in ammonium bicarbonate, and purified using Sephadex G-25 gel filtration (Sigma, India). A schematic diagram of the AMP extraction and purification process is provided in Supplementary Figure S1.

After evaporating trifluoroacetic acid (TFA) and extracting with diethyl ether, the crude peptide was dissolved in H_2_O, lyophilized, and analyzed by HPLC with a semi-preparative C18 column (Pursuit 10C18, Agilent Technologies, USA). The purified peptide was further analyzed by MALDI to determine its amino acid sequence. The lyophilized peptide was then solubilized in double-distilled water to a final concentration of 1 mM, filter-sterilized (0.2 μm, Whatman), and further diluted to 200 ppm for use.

### 2.3 Preparation of culture filtrate from *Lactiplantibacillus spp.*

The flask containing MRS broth inoculated with *Lactiplantibacillus* spp. was incubated at 28°C for 24 h to allow bacterial biomass growth. Subsequently, the flask was placed on a rotary shaker at 150 rpm for 72 h. The culture filtrate, devoid of bacterial cells, was then separated and filtered through Whatman filter paper no. 1 before being concentrated using a rotary evaporator.

### 2.4 Analysis of bacterial cell morphology

The destruction of bacterial cell morphology in *R. solanacearum* caused by the antimicrobial peptide (AMP) was observed through scanning electron microscopy (SEM). Bacterial cells treated with AMP, as well as those from the control group, were first suspended in 2.5% glutaraldehyde in phosphate buffer for 1.5 h to preserve their structure. Following fixation, the cells were thoroughly washed three times with phosphate buffer (0.1 M, pH 7.2) for 5 min each to remove any excess glutaraldehyde.

To further prepare the cells, they were resuspended in osmium tetroxide (OsO₄) for 1 h to enhance contrast, after which they were washed three times with phosphate buffer to remove any residual osmium tetroxide (Zhu et al., 2010). The washing steps ensured that potential contaminants, including remnants of the fixation chemicals, were effectively removed before proceeding with dehydration.

The samples were then dehydrated by passing through a graded ethanol series (30, 50, 70, 90, and 100%) for 10 min at each concentration, followed by treatment with hexamethyldisilazane (HMDS) for 10 min to ensure complete dehydration. Once air-dried, the bacterial cells were coated with gold ions using a sputter coater to avoid any charging effects during imaging, thereby ensuring accurate SEM observation. This thorough preparation effectively removed potential contaminants prior to scanning electron microscopy (SEM) evaluation. Imaging was done in high vacuum mode (Parameswaran and Verma, 2011; Ye et al., 2022).

### 2.5 Tomato growth conditions

Tomato seeds of the desired variety PKM-1 were obtained from Tamil Nadu Agriculture University, India. The soil used in this study was prepared with a composition suitable for tomato growth, consisting of loamy soil with a pH ranging from 6.0 to 6.8. Prior to use, the soil mixture underwent sterilization by heating in an autoclave at 121°C for 30 min to eliminate any potential pathogens and unwanted microorganisms. For the pot experiment, pots measuring 15 cm in height and 12 cm in width were employed to accommodate the tomato plants comfortably. Each pot was filled with approximately 300 g of the prepared soil mixture. Before planting, the soil was adequately moistened to ensure proper hydration for seed germination (Roberts et al., 2005).

Tomato seeds were sown in each pot, with a spacing of approximately 2–3 cm between seeds. The pots were then placed in a controlled environment conducive to tomato seed germination, maintaining optimal temperature and humidity conditions (around 28°C and 70–80% relative humidity) (Vos et al., 2014). Four seeds were added to each pot. The soil composition consisted of peat and soil in a ratio of 1:2. For plant growth promotion studies, each experimental setup was conducted in different sets (plants treated with extracted peptide only (PR1); plants treated with concentrated culture filtrate/supernatant of *Lactiplantibacillus* spp. IT only (SR1); plants treated with bacterial culture *Lactiplantibacillus* spp. IT (BR1) and control), with a total of 20 pots in each experimental setup In biocontrol studies, group of plants were inoculated with *R. solanacearum* (Rs) individually. Throughout the growth period, the tomato plants were regularly irrigated with water to maintain soil moisture levels. Adequate ventilation was provided to prevent the buildup of humidity and reduce the risk of fungal diseases. Regular monitoring of plant growth parameters was conducted to assess the effects of different treatments on plants (Mao et al., 1998; Khan et al., 2020).

### 2.6 Greenhouse experiment of biocontrol effect on severity index of tomato bacterial wilt and plant biomass

The extracted antimicrobial peptide solution, bacterial media supernatant of *L. argentoratensis* strain IT, and cell suspension of *L. argentoratensis* strain IT were utilized for *in vivo* assessment to suppress bacterial wilt in tomatoes. Cell suspensions of *Lactiplantibacillus* spp. (approximately 10^8^ CFU/ml) and *R. solanacearum* were prepared in suitable media on a rotary shaker at 130 rpm and 30°C for 24 h. Seeds of a susceptible variety of tomato plants (PKM-1) obtained from Tamil Nadu Agriculture University, India, were used for the assay. The seeds were sterilized with a 2% sodium hypochlorite solution for 2 min and rinsed three times with sterile water (Bhattacharya et al., 2024). Following this, the seeds were placed onto the surfaces of wet sterilized filter papers and left to incubate at room temperature for 5 days. After germination, the seeds were planted into pots filled with sterilized soil mixtures, and the pots were transferred to a greenhouse with a relative humidity level of 70–80% and temperatures ranging from 25 to 30°C. Following a week of growth, the *Solanum lycopersicum* seedlings were carefully taken out of the pots, washed with tap water, and their roots were excised (wounded) before being submerged for an hour in the *R. solanacearum* cell suspension. They were then put back into the same pots and maintained in the previously mentioned environmental settings. The control treatment consisted of the roots submerged in sterile water as well as inoculation with only pathogen. After 14 days, the prepared suspensions of culture supernatant of *Lactiplantibacillus* spp. IT (SR1), cell suspension of *Lactiplantibacillus* spp. IT (BR1), and microbial peptide suspension (PR1) were sprayed on the plants to observe the biocontrol effect of the selected treatment on the severity index of bacterial wilt. Each treatment was assigned to 20 plants, and each treatment was carried out in three independent sets. On a daily basis, the seedlings were observed, and any symptoms or changes were carefully noted. Thirty days after treatment, the disease’s incidence, severity, and effectiveness to control BW were assessed using a 5-point grading system: 0 represented no symptoms, 1 one partially wilted leaf, 2 two or three wilted leaves, 3 all wilted leaves except the top two or three, 4 all wilted leaves, and 5 the plant was dead. Disease severity (%) was calculated as (disease ratings × number of diseased plants)/(maximum rating value × total number of plants) × 100; disease incidence (%) was calculated as (number of diseased plants/total number of plants investigated) × 100; and biological control efficacy (%) was calculated as (wilt incidence of control × wilt incidence of treatment/wilt incidence of control) × 100. Furthermore, measurements of plant lengths and fresh and dry weights were made 30 days after treatment to evaluate the effect of various strains on the biomass of *Solanum lycopersicum* plants (Gupta et al., 2024).

### 2.7 Assessment of plant growth promotion and morphological parameters

To evaluate the plant growth promotion (PGP) potential of the biosynthesized microbial peptide, along with *L. argentoratensis* IT strain and its culture filtrate, the PGP assay was conducted in greenhouse conditions, excluding the presence of the plant pathogen *R. solanacearum.* Seeds and seedlings underwent various treatments: they were immersed in cell suspensions prepared for different treatments (bacterial culture supernatant, cell suspension of *Lactiplantibacillus* spp. IT, microbial peptide suspension) for 8 h, followed by air-drying in a laminar flow hood. Inoculation with sterile water served as the control (Mahreen et al., 2023). Each treatment consisted of 20 plants, with three independent replicates conducted for each treatment. During the same growth period (30 days after the spray treatment, as described above), various physical characteristics were measured, including shoot length, root length, root fresh/dry weight, and shoot fresh/dry weight. To ascertain the dry weights of plant organs, they underwent a drying process in an oven at 60°C for a duration of 3 days. The growth promotion efficacy (GPE %) was then calculated using the formula: GPE % = [(treatment – control)/control] × 100 (Bhuimbar and Dandge, 2022).

## 3 Biochemical parameters of plants

### 3.1 Total chlorophyll content

Plant biochemical parameters were analyzed, including the total chlorophyll content. Chlorophyll estimation followed a modified method based on protocols by Lichtenthaler (1987), Porra et al. (1989), and Wellburn (1994). In this method, 1 g of leaf tissue was submerged in a 10 mL solution of 80% acetone and left to incubate for 24 h in darkness to aid in pigment extraction. Subsequently, the absorbance of the resulting green solution was measured at 662 and 645 nm using a UV–Vis spectrophotometer. Total chlorophyll content was determined employing the formula proposed by Lichtenthaler (1987), with pigment quantities expressed in micrograms per gram fresh weight (μg/g FW) (Sudalai et al., 2013). Chlorophyll a (Chl a) concentration was computed as [(ABS662 × 11.75) – (ABS645 × 2.35)], while Chlorophyll b (Chl b) was calculated using [(ABS645 × 18.61) – (ABS662 × 3.96)]. The total chlorophyll content was then obtained by summing the concentrations of Chl a and Chl b (Lichtenthaler and Buschmann, 2001).

### 3.2 Flavonoids content

The flavonoid content was determined using the method described by Liang et al. (2013), with slight modifications. The extracts were dissolved in absolute methanol. In a 2 mL Eppendorf tube, 100 μL of a sample was mixed with 430 μL of 5% NaNO_2_, followed by incubation for 5 min. After incubation, 30 μL of AlCl_3_ (10%) and 440 μL of NaOH (1 mol/L) were added to the reaction mixture, and the absorbance was read at 496 nm with a multimode spectrophotometer (BMG Labtech, Chicago, IL, USA), using quercetin as the standard. The results were expressed as mg of quercetin equivalents (QE) per g of extract (mg QE/ge) (Oikeh et al., 2020).

## 4 Analysis of defense-related enzymatic activities

### 4.1 Assessment of superoxide dismutase (SOD) activity

Superoxide dismutase (SOD) activity was measured spectrophotometrically at 560 nm by assessing the inhibition of nitro blue tetrazolium chloride (NBT) photoreduction. A crude enzyme extract (50 μL) was combined with a reaction mixture containing 2.5 mL of 0.1 M potassium phosphate buffer (pH 7.8), 75 μM riboflavin, 2 mM NBT, 3 mM EDTA, and 200 mM methionine. The reaction mixture was exposed to white light from a 25 W fluorescent lamp for 15 min at 25°C. A control solution (without enzyme) was kept in darkness (Haida and Hakiman, 2019). The absorbance increase due to formazan formation at 560 nm was recorded, and enzyme activity was calculated as the percentage inhibition of photoreduction, with 100% representing the positive control without enzyme extract. SOD activity was expressed in units (U) per gram fresh weight, with one unit of SOD defined as the amount of enzyme required for 50% inhibition of NBT photoreduction (Beyer and Fridovich, 1987).

### 4.2 Assessment of peroxidase (POD) activity

Peroxidase (POD) activity was measured by monitoring the oxidation of guaiacol, a substrate, at 470 nm. The reaction mixture contained 100 mM potassium phosphate buffer (pH 7.0), 1% guaiacol, and 3% hydrogen peroxide. To 2.5 mL of this solution, 50 μL of crude enzyme extract was added, and the absorbance was recorded at 30-s intervals over a 5-min period. POD activity was expressed as micromoles of guaiacol oxidized per minute per milligram of protein, using a molar extinction coefficient of 26.6 mM^−1^ cm^−1^ (Allain et al., 1974).

### 4.3 Assessment of lipoxygenase (LOX) activity

Lipoxygenase (LOX) activity was evaluated using 100 mM potassium phosphate buffer (pH 6.5) and 10 mM linoleic acid as the substrate. To 2.5 mL of this reaction mixture, 50 μL of crude enzyme was added. Absorbance was measured at 234 nm, every 30 s for 5 min, as the enzyme catalysed the oxidation of linoleic acid. The increase in absorbance corresponded to LOX activity, which was expressed in enzyme units (Gardner, 2001).

### 4.4 Assessment of polyphenol oxidase (PPO) activity

Polyphenol oxidase (PPO) activity was determined by measuring the oxidation of catechol, a common substrate, at 420 nm. The reaction mixture contained 100 mM potassium phosphate buffer (pH 6) and 0.1 M catechol. To 2.5 mL of the mixture, 50 μL of crude enzyme extract was added. Absorbance was recorded at 30-s intervals over a 4-min period. PPO activity was expressed as micromoles of product formed per minute per milligram of protein, using a molar extinction coefficient of 1,300 M^−1^ cm^−1^ (Sikora et al., 2019; Chin et al., 2020).

### 4.5 Assessment of catalase (CAT) activity

Catalase (CAT) activity was measured by monitoring the degradation of hydrogen peroxide (H_2_O_2_) at 240 nm. The reaction mixture contained 100 mM potassium phosphate buffer (pH 7) and 20 mM H_2_O_2_. To 2.5 mL of this mixture, 50 μL of crude enzyme extract was added. The reduction in absorbance was recorded at 240 nm, and CAT activity was expressed as the amount of H_2_O_2_ decomposed per minute per milligram of protein, using a molar extinction coefficient of 0.036 mM^−1^ cm^−1^ (Shangari and O’Brien, 2006).

### 4.6 Estimation of total protein content

To determine the protein concentration in the extracts, 100 μL of protein extract containing around 10–100 μg was utilized. This extract was thoroughly mixed with 5 mL of dye reagent. Simultaneously, a series of standards was prepared using Bovine Serum Albumin (BSA) from a 2.0 mg/mL stock solution in extraction buffer. Each standard solution, ranging from 5 to 100 μL of BSA, was brought to a final volume of 100 μL with extraction buffer and then mixed with 5 mL of dye reagent by vortexing. After a 5-min incubation period, the absorbance was measured at 595 nm (OD_595_) for both the extracts and standards against a reagent blank containing 100 μL of extraction buffer with 1 mL of dye reagent. Final protein concentration was determined in extract by comparing the absorbance readings with the standard curve for BSA. If the OD_595_ for the diluted extract fell outside the acceptable range, a more appropriate dilution was prepared for accurate quantification (Baerlocher, 2020).

### 4.7 Antioxidant estimation by phosphomolybdate assay

The phosphomolybdate assay was performed following the procedure where, initially, 0.05 g of ground tissue was placed into a cooled Eppendorf tube, followed by the addition of 1 mL of the specified solvent according to the selected protocol. After briefly vortexing for homogenization, the mixture was allowed to incubate overnight (12–16 h) at a designated temperature, typically 4°C or − 20°C. Subsequent steps, such as centrifugation and collection of the supernatant, were performed according to the specific requirements of the assay. The resultant mixture was then prepared by combining a predetermined volume of the extract with the reagent solution, followed by thorough mixing. Incubation conditions, including temperature and duration, were maintained as per the assay protocol. After the prescribed incubation period, the absorbance reading of the reaction solution was noted at the specified wavelength using a spectrophotometer. The total antioxidant capacity was measured and reported accordingly (Jan et al., 2013).

## 5 Results

### 5.1 Effect of different treatment on tomato bacterial wilt

Various treatments were deployed to assess biocontrol activity against tomato bacterium wilt (BW) in form of foliar spray, with the antimicrobial peptide (AMP) derived from *Lactiplantibacillus* spp. IT (PR1 + RS1), culture filtrate (SR1 + RS1), bacteria cell culture (BR1 + RS1) showing suppression of the disease. Treatments effectively delayed symptoms and reduced disease incidence and severity. Ten days post-inoculation, foliage symptoms were observed on control set of plants infected with *R. solanacearum* (BI0001). Sixteen days after the foliar spray application, foliage symptoms emerged on plants subjected to co-treatment with BR1 + RS1. Twenty days after treatment, foliage symptoms were evident on plants co-treated with PR1 + RS1 and SR1 + RS1, with greater severity observed in plants inoculated with BR1 + RS1 compared to PR1 + RS1 and SR1 + RS1. Although all formulations significantly inhibited bacterial wilt compared to the pathogen control (*p* < 0.05), the spray formulation containing AMP (PR1 + RS1) demonstrated the highest control efficacy at 55.1%. This indicated a significant decrease in disease incidence and severity. Specifically, disease incidence decreased by 49.3%, and disease severity decreased by 45.8%. Following this, the control efficacy was 37.9% in SR1 + RS1 and 24.1% in BR1 + RS1. As time progressed, control efficacy increased, while disease incidence and severity decreased across different treatments. Moreover, the experiments also confirmed *R. solanacearum* as the causative agent of tomato bacterial wilt (Table 1).

**Table 1:** Biocontrol efficacy of different sets of treatment against tomato bacterium wilt after 30 days in presence of *Ralstonia spp.*

Treatment	Disease severity	Disease incidence	Control efficacy
SR1 + RS1	34.0 ± 2.3^d^	60.0 ± 2.5^c^	37.9
PR1 + RS1	50.6 ± 1.0^c^	43.3 ± 2.0^d^	55.1
BR1 + RS1	69.3 ± 1.9^b^	73.3 ± 1.3^b^	24.1
Control	96.0 ± 2.3^a^	80.0 ± 2.5^a^	–

Each value is a mean of three replicates from three experiments. Mean ± SD followed by the same letter in a column of each treatment is not a significant difference at *p* = 0.05 by the Tukey–Kramer HSD test (RS1: *Ralstonia solanacearum*, SR1: Supernatant, BR1: Bacteria culture, PR1: Peptide suspension).

Each value is a mean of 3 replicates from each experiment. Mean ± SD followed by the same letter in a column of each treatment is not a significant difference at *p* = 0.05 by the Tukey–Kramer HSD test.

The impact of different foliar spray treatments on plant growth biomass was assessed under varying conditions of pathogen stress. The collected data demonstrated significant effects of the formulated suspension spray of microbial peptide on growth and biomass accumulation compared to the pathogen controls (*p* < 0.05), as depicted in Table 2. Treatment with PR1 + RS1 led to the greatest increases in root and plant lengths by 53.66 and 91.67% also seedling and root dry weight by 80.73 and 40.04%, respectively among the treatments.

**Table 2:** Assessing the impact of various treatments on tomato plant growth promotion in presence of phytopathogen (RS1: *Ralstonia solanacearum*, SR1: Supernatant, BR1: Bacteria culture, PR1: Peptide suspension).

Plant	Plant length	GPE (%)	Root length	GPE (%)	Seedling fresh weight	GPE (%)	Seedling dry weight	GPE (%)	Root fresh weight	GPE (%)	Root dry weight	GPE (%)
SR1 + RS1	35.21 ± 1.32^b^	71.8	7.12 ± 0.01^b^	47.10	15.39 ± 1.10^a^	77.5	3.91 ± 0.110^b^	76.12	0.81 ± 0.01^b^	26.56	0.13 ± 0.01^a^	30
PR1 + RS1	40.12 ± 1.54^a^	94.13	9.52 ± 0.13^a^	96.69	15.87 ± 1.56^a^	80.73	4.21 ± 0.02^a^	89.6	0.85 ± 0.02^a^	32.8	0.14 ± 0.05^a^	40.0
BR1 + RS1	25.85 ± 0.72^c^	25.97	6.58 ± 0.01^c^	35.95	8.96 ± 0.70^b^	3.44	2.30 ± 0.04^c^	3.60	0.80 ± 0.00^b^	25.01	0.10 ± 0.09^a^	0.0
Control	20.52 ± 0.12^d^	–	4.84 ± 0.11^d^	–	8.67 ± 0.72^b^	–	2.22 ± 0.03^d^	–	0.64 ± 0.06^c^	–	0.1 ± 0.01^a^	–

Each value is a mean of three replicates from three experiments. Mean ± SD followed by the same letter in a column of each treatment is not a significant difference at *p* = 0.05 by the Tukey–Kramer HSD test.

### 5.2 Assessment of plant growth promotion and morphological parameters

**Figure 1:**
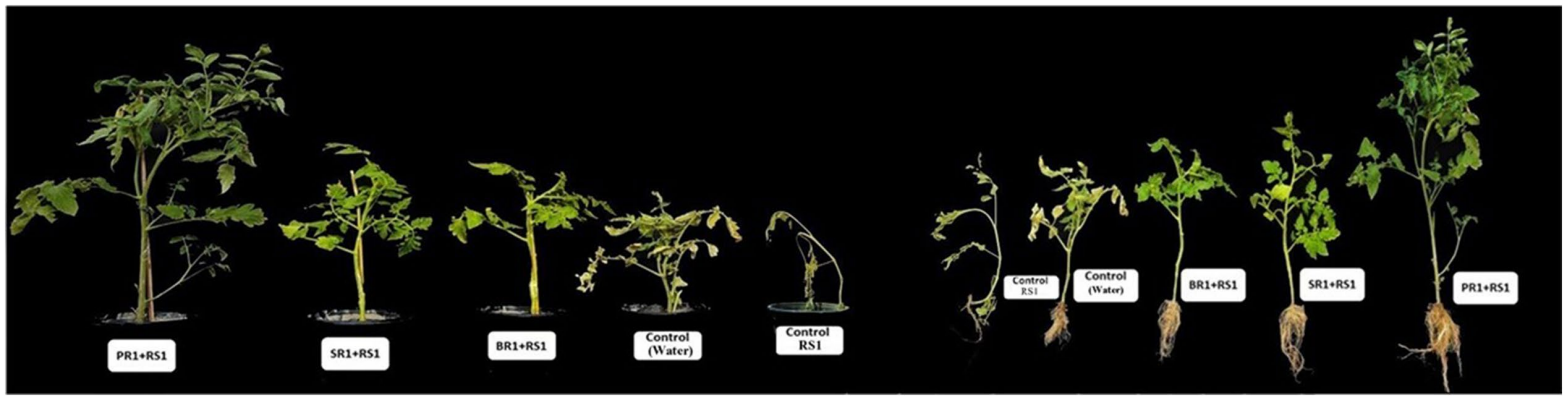
Efficacy of foliar spray application on growth promotion of *Solanum lycopersicum* PKM-1 in presence of *Ralstonia solanacearum* (RS1: *Ralstonia solanacearum*, SR1: Supernatant, BR1: Bacteria culture, PR1: Peptide suspension).

Plant growth-promoting (PGP) tests were carried out, recording a variety of physical parameters like root and plant length, fresh and dry weight of the shoots, and fresh and dry weight of the roots in order to evaluate the effectiveness of PGP treatments in a pathogen-free environment (Table 3). Overall, the highest plant growth was observed in plants set with a sequential application of antimicrobial peptide (PR1), resulting in 96.23% increase in shoot length. Furthermore, plants treated with culture filtrate (SR1) and bacterial cell culture (BR1) showed respective increases in root length of 66.26 and 23.41% (Figure 1). When treated with a sequential spray of AMP (PR1), culture supernatant (SR1), and bacterial culture of *Lactiplantibacillus* spp. (BR1), the fresh and dry weight of roots were 39.3, 12.7, and 2.12% higher when compared to control plants.

**Table 3:** Assessing the impact on plant growth promotion of tomato plant in absence of phytopathogen (RS1: *Ralstonia solanacearum*, SR1: Supernatant, BR1: Bacteria culture, PR1: Peptide suspension).

Plant	Plant length	GPE (%)	Root length	GPE (%)	Seedling fresh weight	GPE (%)	Seedling dry weight	GPE (%)	Root fresh weight	GPE (%)	Root dry weight	GPE (%)
SR1	46.62 ± 1.13^b^	75.5	15.13 ± 0.13^b^	66.2	18.67 ± 0.13^ab^	16.6	5.14 ± 0.23^a^	19.2	1.06 ± 0.02^b^	12.7	0.16 ± 0.01^b^	14.28
PR1	52.03 ± 1.21^a^	95.7	17.52 ± 0.15^a^	92.3	19.13 ± 1.73^a^	19.4	5.25 ± 0.15^a^	21.8	1.31 ± 0.01^a^	39.3	0.21 ± 0.01^a^	49.9
BR1	37.89 ± 1.24^c^	42.6	11.23 ± 0.73^c^	23.40	18.34 ± 1.15^b^	14.5	4.81 ± 0.23^b^	11.6	0.96 ± 0.01^c^	2.12	0.16 ± 0.01^b^	14.28
Control	26.56 ± 1.73^d^	–	9.10 ± 0.01^d^	–	16.01 ± 1.13^c^	-	4.31 ± 0.11^c^	–	0.94 ± 0.02^d^	–	0.14 ± 0.01^c^	–

Each value is a mean of three replicates from three experiments. Mean ± SD followed by the same letter in a column of each treatment is not a significant difference at *p* = 0.05 by the Tukey–Kramer HSD test.

The results indicated that the spray formulation significantly enhanced biomass and growth accumulation compared to the controls (p < 0.05). Notably, the spray treatment with microbial peptide PR1 yielded the highest plant and root lengths, increasing by 95.9 and 92.5%, respectively, as well as the root dry weight and seedling weight, increasing by 49.9 and 21.8%, as shown in Table 3. Similarly, the fresh and dry weight of shoot samples were 20 and 23% higher respectively, in plants treated with a sequential spray of microbial peptide (PR1) as compared to control set of plants.

### 5.3 Impact of microbial peptide, culture filtrate, and *Lactiplantibacillus* spp. on the biochemical parameters of *Solanum lycopersicum* PKM-1

**Figure 2:**
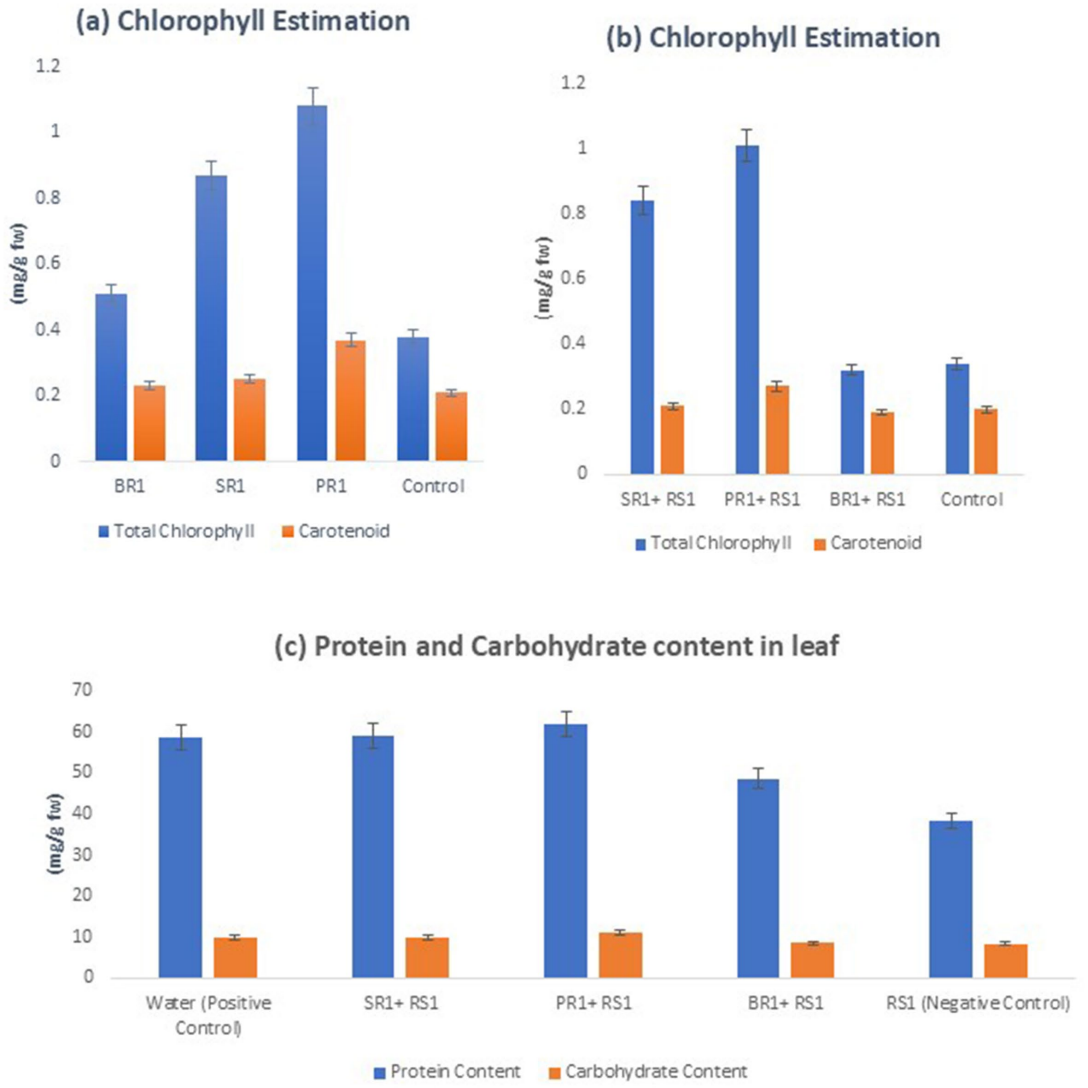
**(A)** Effect of different treatment on chlorophyll content in absence of phytopathogen **(B)** Effect of different treatment on chlorophyll content in presence of phytopathogen **(C)** Effect of different treatment on protein and carbohydrate content in leaf (RS1: *Ralstonia solanacearum*, SR1: Supernatant, BR1: Bacteria culture, PR1: Peptide suspension). *Values represent the mean ± SD (standard deviation) of three independent experiments for each treatment.

Biochemical parameters, including chlorophyll and protien content, were measured (Figures 2A–C). The highest levels were observed in plants treated with microbial peptide spray (PR1), followed by those treated with culture supernatant (SR1) and *Lactiplantibacillus* spp. IT cell culture (BR1). Chlorophyll content, measured in mg/g fresh weight, exhibited a more pronounced increase in plants treated with microbial peptide spray (PR1), showing a rise of approximately 73.81%, compared to plants treated with culture filtrate (SR1), which showed an increase of about 52.38%. Similarly, total soluble sugar, measured in mg/g, showed a greater increase in plants treated with microbial peptide spray compared to those treated with individual *L. argentinontensis* cell culture.

**Figure 3:**
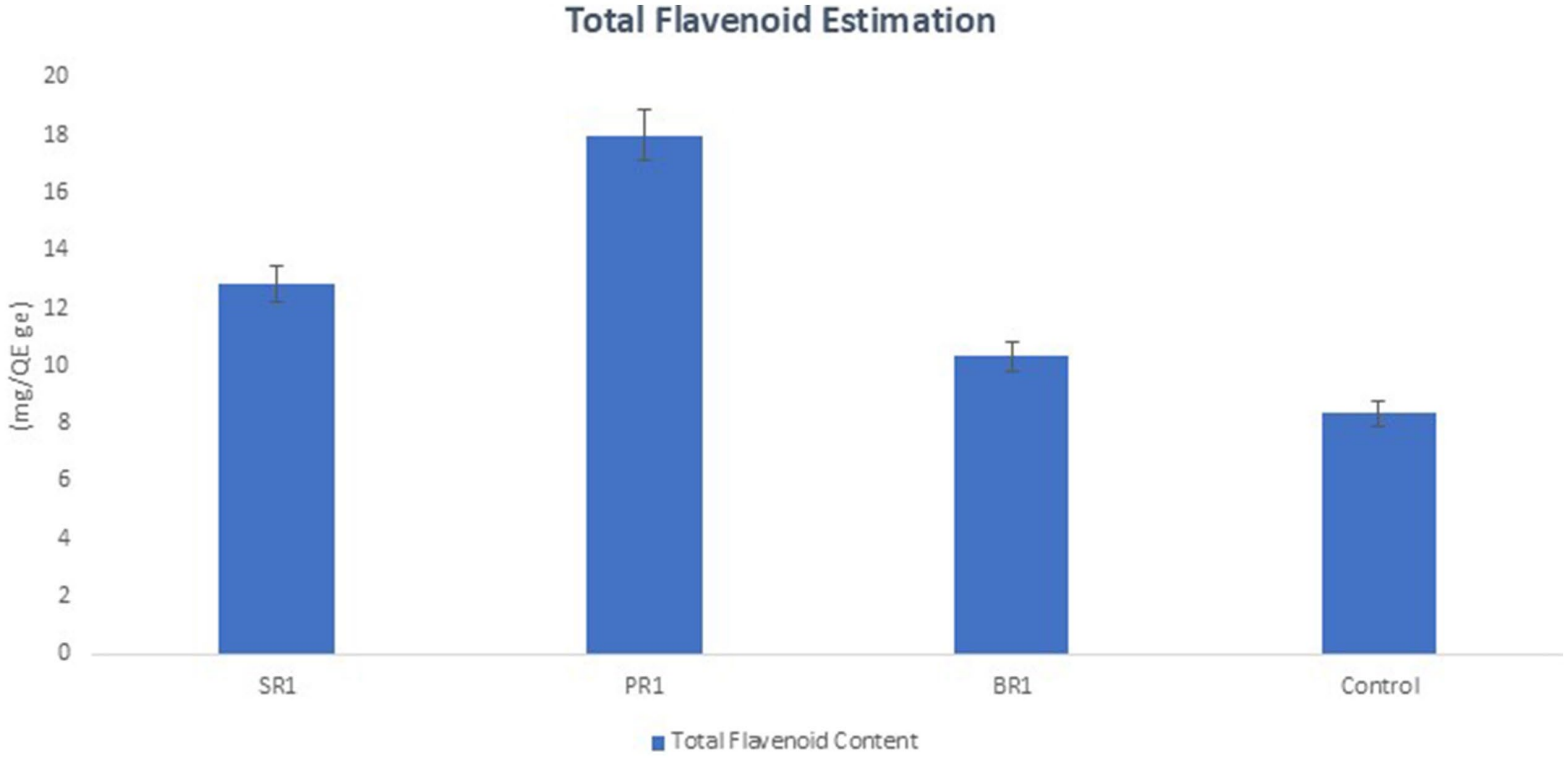
Effect of foliar spray on total flavonoid content in leaves of *Solanum lycopersicum* (RS1, *Ralstonia solanacearum*; SR1, Supernatant; BR1; Bacteria culture; PR1, Peptide suspension). *Values represent the mean ± SD (standard deviation) of three independent experiments for each treatment.

Flavonoid content was assessed in terms of mg of quercetin equivalents per g of extract as shown in Figure 3. It showed an increase in all treated plants, with a more substantial increase observed in plants treated with a sequential spray of microbial peptide (115.95%) and culture filtrate spray of *L. arentinonsis* (53.96%) compared to plants treated with cell culture of *L. argentinotensis* (23.74%).

### 5.4 Defense enzyme activity estimation in tomato plants

#### 5.4.1 Peroxidase activity (PO)

**Figure 4:**
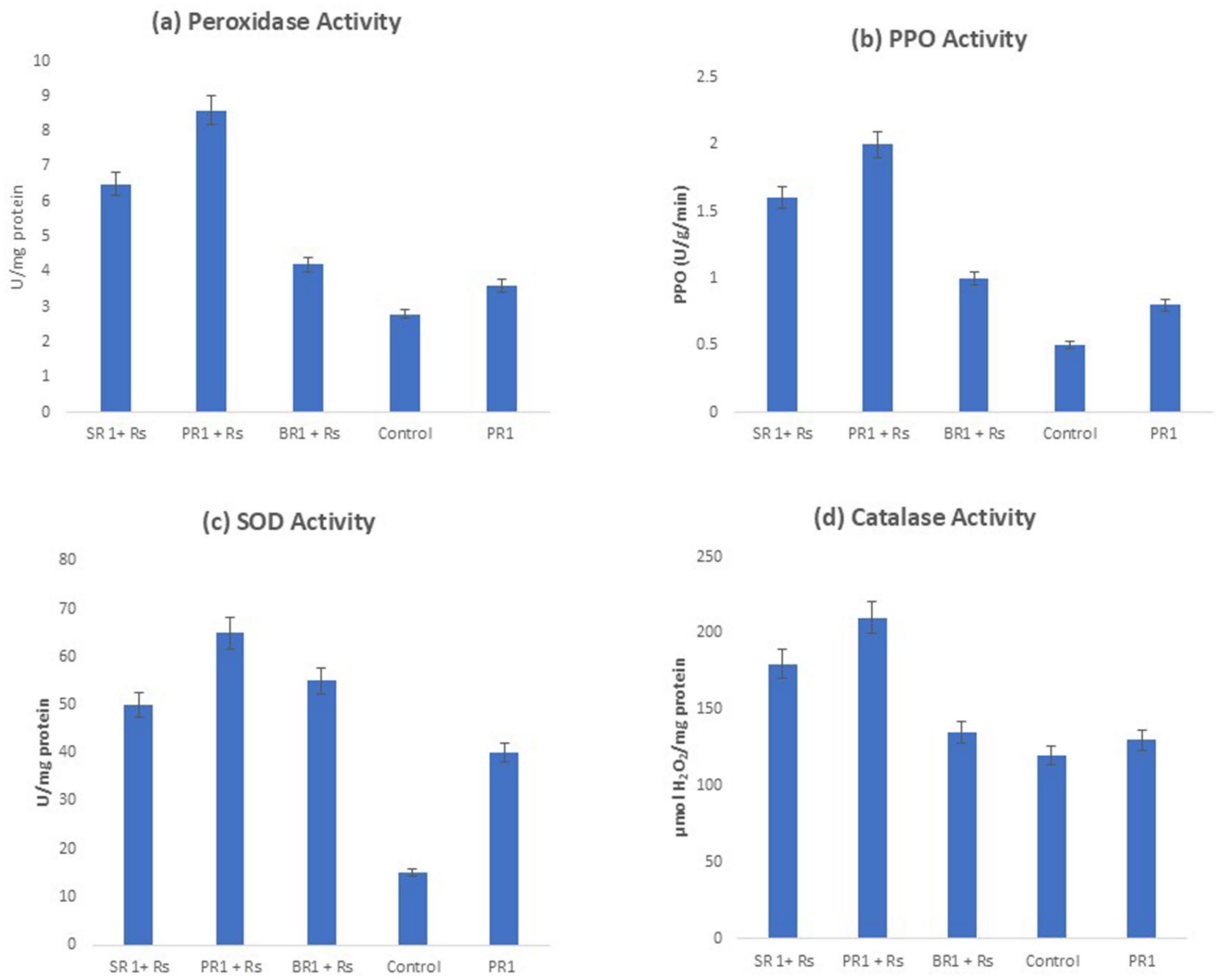
Estimation of various defense enzymes including **(A)** Peroxidase Activity **(B)** Polyphenol Oxidase **(C)** Superoxide Dismutase **(D)** Catalase Activity when treated with foliar spray of microbial peptide (PR1 + Rs), culture filtrate of *Lactiplantibacillus spp.* (SR1 + Rs), cell culture of *Lactiplantibacillus spp.* (BR1 + Rs) *Rs, *Ralstonia solanacearum.* *Values represent the mean ± SD (standard deviation) of three independent experiments for each treatment.

In plants, the activities of peroxidase were observed across different treatment sets. Among these treatments, the application of microbial foliar spray (PR1), followed by the spray of culture filtrate of *Lactiplantibacillus* spp., recorded the highest induction of peroxidase activities (8.6 U/mg). Increases in enzyme activity were noted in all treatments. Tomato plants that received no treatment showed the lowest induction of peroxidase activity compared to all other treatments (Figure 4A).

#### 5.4.2 Polyphenol oxidase (PPO)

Under greenhouse conditions, the application of microbial spray induced a higher level of PPO activity (2 U/mg), subsequently, the plants were sprayed with the culture filtrate also showed considerable increase in PPO activity (1.6 U/mg). The control set plants exhibited the minimum PPO activity as compared to all other treatments (Figure 4B) (Hadwan et al., 2024).

#### 5.4.3 Assessment of superoxide dismutase (SOD) activity and catalase activity (CAT)

Reactive oxygen species play a significant role in tissue degradation under both abiotic and biotic stress conditions. Regarding superoxide dismutase (SOD) activity, plants treated with microbial peptides (PR1 + RS1) exhibited the maximum increase, reaching 65 U/min/mol, which was the highest among all treatment groups as shown in (Figure 4C). On the contrary, plants treated with culture filtrate (SR1 + RS1) exhibited a measured value of 31.7 U, which ranked as the second highest among the plant groups, in comparison to the control. In the plant group set (BR1 + SR1), a decline in SOD activity to 50 U/min/mol was observed upon spraying. Moreover, the elevated SOD activity observed in plants treated with microbial peptide suggests non-toxic antioxidant scavenging activity in plants.

Tomato plants treated with microbial peptide (PR1 + RS1) exhibited a notably higher percentage of CAT activity around 75%. This increase could be attributed to the biocontrol effect of stress, leading to enhanced O2- accumulation (Figure 4D). Additionally, plants sprayed with culture filtrate (SR1 + RS1) also exhibited a significant increase of 50% in SOD activity compared to plants treated with (BR1 + RS1), which showed lower SOD activity, indicating downregulation of enzymes.

### 5.5 Electron microscopy analysis of *Ralstonia solanacearum* cells exposed to microbial peptide

**Figure 5:**
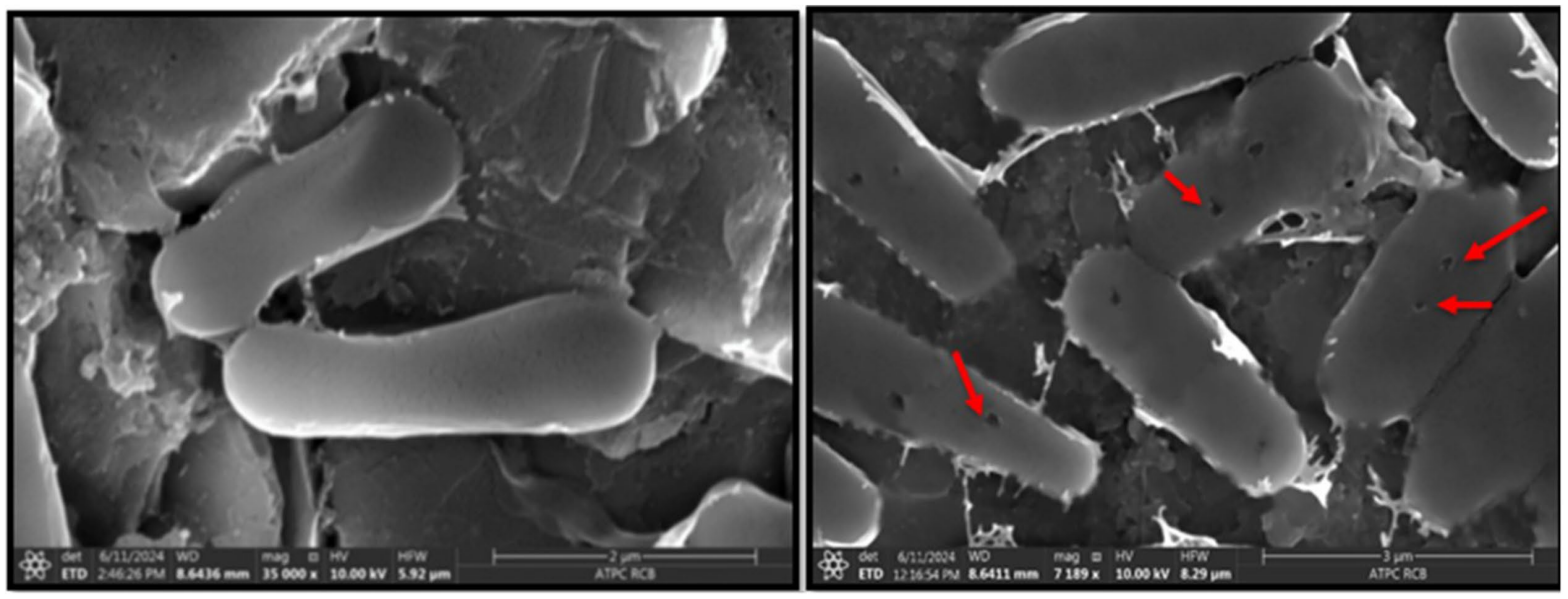
The SEM images reveal pore formation and cell membrane damage in *Ralstonia solanacearum* bacteria after treatment with the microbial peptide, in contrast to the untreated control. The damage is highlighted by arrow indicators.

The interaction between *R. solanacearum* and extracted microbial peptide and the resulting changes in cell morphology and structure were studied using Scanning Electron Microscopy (SEM). Representative SEM images for bacteria species, with and without exposure (control) to microbial peptide are presented in Figure 5.

For *R. solanacearum*, the untreated bacteria appeared as intact bacillus, showing no signs of cell wall rupture or collapse (Control). In contrast, following exposure to microbial peptide, bacteria exhibited extensive pore formation and cell wall damage likely leading to cell lysis, as indicated by the arrows in Figure 5, confirming the mode of action of microbial peptide as Barrel-Stave Model (Lee et al., 2015).

Approximately 38.46% of the cells showed damage after treatment with the microbial peptide.

In the case of untreated *R. solanacearum*, the rod-shaped bacteria maintained an almost intact surface structure. However, after treatment with microbial peptide cells displayed significant changes: they became rougher, with damaged wrinkles and groove-like rifts appearing on the surface. The cell morphology was notably altered, as shown in Figure 5.

## 6 Discussion

Globally, alternative methods for controlling plant diseases and pests are under development to lessen dependence on synthetic agricultural pesticides, thus safeguarding human health and the environment from the harmful effects of these chemicals (Ye et al., 2022; Verma et al., 2017; Yogev et al., 2009; Yuliar et al., 2015). Utilizing naturally produced microbial products is one such approach. Previous studies have demonstrated the efficacy of microbe-derived natural products in reducing plant diseases (Sani et al., 2024; Deb and Tatung, 2024). Among microbes, bacteria provide a diverse range of bioactive compounds and have become a promising group for the exploration and development of novel antimicrobial metabolites (Chakchouk-Mtibaa et al., 2024). *Lactiplantibacillus* spp. has demonstrated the ability to produce a variety of enzymes and bioactive compounds (Ou et al., 2024).Using microbial peptides in agriculture proves to be an effective strategy for boosting both plant yield and resilience against abiotic and biotic stresses (Zhang et al., 2023). Microbial peptides produced by bacteria *Lactiplantibacillus* spp. have garnered significant attention. Their wide-ranging efficacy against bacteria, fungi, viruses, mycoplasma, and tumor cells, combined with their stability, low toxicity to humans and animals, and environmentally friendly characteristics, render them particularly remarkable upon application, AMP may bind to receptor proteins on the plant cell surface, similar to pathogen-associated molecular patterns (PAMPs), which initiate PAMP-triggered immunity (PTI) (Johnson and Temple, 2013; Kurabachew and Wydra, 2013). This interaction likely stimulates a series of downstream signaling pathways, activating transcription factors that regulate the expression of defense-related genes, including those encoding enzymes such as peroxidases, chitinases, and phenylalanine ammonia-lyase. The increase in these enzymes contributes to strengthening the cell wall, detoxifying reactive oxygen species (ROS), and synthesizing antimicrobial secondary metabolites, thereby enhancing the plant’s resilience against pathogen invasion (Tang et al., 2023) Swift reactions to plant pathogens can activate systemic signaling pathways, bolstering plant resistance against pathogen outbreaks (Holaskova et al., 2015).

In this study, a bioactive microbial peptide (PRKGSVAKDVLPDPVYNSKLVTRLINHLMIDGKRGK) derived from *Lactiplantibacillus* spp. was investigated as a biocontrol agent against *R. solanacearum* to enhance resistance to both biotic stress in tomato, one of the major crops cultivated worldwide. The microbial peptide, previously extracted, exhibited the highest antibacterial activity against *R. solanacearum* (Tiwari et al., 2024). However, in this study peptide application as foliar spray was effective against *R. solanacearum*. SEM analysis results unequivocally showcased the membrane-damaging impact of the microbial peptide on *R. solanacearum*. Multiple studies have documented the antimicrobial effects of peptides against diverse pathogenic microbes like *Bacillus megaterium*, *Alternaria alternate*, *Escherichia coli*, and *S. aureus* (Hazarika et al., 2022; Asif et al., 2024). This antibacterial outcome can be attributed to the higher permeability of the microbial peptide to pathogen cell membranes, leading to membrane rupture and protein alterations. This leads to membrane rupture and changes in proteins, disrupting cellular metabolism and ultimately causing the death of bacterial cells (Zhong et al., 2023; Ye et al., 2022). The scanning electron microscopy (SEM) findings from our research provides further confirmation of this antibacterial mechanism.

Results from plant experiments demonstrated that the application of microbial peptide substantially increased the expression of defense-related enzyme activity in tomato plants, effectively suppressing the population of *R. solanacearum*. As a result, bacterial wilt disease severity was reduced, and an increase in plant growth was observed. Microscopic comparisons between vascular tissues from infected and peptide-treated plants also confirmed the peptide’s activity within the vascular system, which is critical for combating the vascular pathogen *R. solanacearum* consistent with previous findings (Yuan and Wang, 2021). Plant defense mechanism can be improved by using exogenous compounds in the form of plant- or microbe-based natural compounds. Bacteria offer a rich source of natural antimicrobial compounds which may serve as natural elicitors, inducing host resistance to improve plant defense strategy against several pathogens. Several antimicrobial peptides have been previously isolated from bacteria of the *Lactobacillus* genus, showing efficacy against soil-borne pathogens (Gilliard et al., 2024; Song et al., 2021; Li et al., 2023; Saikia et al., 2022). These studies have also reported improved plant growth in various crops. Above studies indicates that microbial peptide as well as concentrated culture filtrate of *Lactiplantibacillus* spp. (PRKGSVAKDVLPDPVYNSKLVTRLINHLMIDGKRGK) has a good potential in the form of biocontrol agent and plant growth promoter for *Solanum lycopersicum* crop. While this study demonstrates the effectiveness of antimicrobial peptides (AMPs) under controlled greenhouse conditions, several challenges may arise during field application. Peptide stability is a significant concern, as environmental factors such as UV exposure, temperature fluctuations, and varying humidity levels can potentially degrade the peptide’s structure and efficacy. While AMPs offer promising environmental benefits as alternatives to synthetic pesticides, several limitations need consideration. A primary challenge is the rapid degradation of AMPs when exposed to environmental factors such as UV light, temperature fluctuations, and varying humidity levels. This degradation can reduce their efficacy in open-field conditions, suggesting a need for formulations that enhance peptide stability. Additionally, while AMPs generally exhibit low toxicity to humans and animals, their effects on non-target organisms, including beneficial soil microbes and pollinators, remain an area for further investigation. Potential impacts on these organisms could influence soil health and broader ecosystem dynamics. Additionally, the formulation of AMPs for field use requires advanced delivery systems to enhance stability and ensure sustained release. Encapsulation techniques, such as nano- or microencapsulation, present promising options to protect AMPs from environmental degradation and provide controlled release over time.

The application of culture filtrate of bacteria can reduce the wide ranging of antimicrobial compound required for certain field broadcasting since microbial peptides are extracellular in nature (Pradhanang et al., 2005; Siddique et al., 2020; Srinivasamurthy et al., 2014). Culture filtrate is cost-effective and requires less manpower for production. The use of microbial peptide derived from *Lactiplantibacillus* spp. shows significant promise as a key component in integrated disease management strategies for bacterial wilt. To effectively integrate antimicrobial peptide (AMP) into current integrated pest management (IPM) strategies, AMP can be used as biocontrol agents alongside traditional methods. AMP can be applied as foliar sprays or soil treatments to enhance plant resistance against bacterial wilt while reducing reliance on synthetic pesticides. Additionally, combining AMPs with reduced rates of conventional pesticides may improve efficacy and sustainability in pest control. Monitoring the impact of AMP on non-target organisms and overall ecosystem health is essential, alongside educational outreach to inform farmers about their benefits. This comprehensive approach underscores the potential of AMP to significantly enhance existing pest management programs in tomato cultivation. Being a natural product, it is environmentally friendly and presents challenges for pathogens to develop resistance against it (Tan et al., 2024).

## 7 Conclusion

This study provides a comprehensive evaluation of a novel antimicrobial peptide (AMP) derived from *L. argentoratensis* strain IT, demonstrating its potential as an effective biocontrol agent against *R. solanacearum*, the causative pathogen of bacterial wilt in tomato plants. Our findings reveal that the AMP not only exhibited potent antibacterial properties but also induced significant morphological damage to the bacterial cells, as confirmed by scanning electron microscopy (SEM). Importantly, the application of the AMP as a foliar spray enhanced tomato plant resistance by upregulating defense-related enzymes and stimulating key physiological improvements, including increases in plant height, root length, photosynthetic pigments, and protein content. The novelty of this research lies in the dual action of the AMP—providing both direct antimicrobial effects and promoting the plant’s own defense mechanisms. This study is the first to demonstrate the successful use of a foliar-applied microbial peptide against *R. solanacearum*, presenting a potential breakthrough in bacterial wilt management. Additionally, this approach showed significant reductions in disease incidence and severity while enhancing overall plant growth and resilience.

Furthermore, the study highlights the AMP’s ability to stimulate defense responses while being an environmentally friendly alternative to synthetic pesticides. Numerous studies have shown that the activation of specific defense enzymes, such as peroxidases, chitinases, and phenylalanine ammonia-lyase, is closely linked to the transcriptional regulation of corresponding defense genes. This correlation supports the idea that increased enzyme activity reflects the underlying activation of these genes, thus providing insight into the plant’s physiological responses to pathogen attack. While our current approach primarily emphasizes enzyme activity estimation for gene expression, there is also scope of more comprehensive understanding of the molecular mechanisms at play which would benefit from the integration of quantitative PCR (qPCR) analyses in future research. This combination of antimicrobial and plant-growth-promoting effects makes the AMP a promising candidate for integrated pest and disease management strategies in tomato cultivation. As agriculture shifts toward more sustainable and eco-friendly practices, microbial peptides like the one investigated here hold immense potential. By reducing reliance on chemical pesticides and contributing to enhanced crop protection, this study offers a foundation for future research aimed at refining the use of microbial peptides in large-scale agricultural systems. The findings underscore the need for continued exploration of microbial peptides as natural, sustainable solutions to agricultural challenges, with the ultimate goal of improving crop yield, food security, and environmental sustainability.

## 8 Statistical analysis

Standard deviation for all experiments was calculated using Microsoft Excel. To evaluate the significance of the results across different treatments, statistical analysis was performed using JMP software version 11 (SAS Institute, Inc., Cary, NC). The significance of differences between treatments was determined using the Tukey–Kramer HSD test, with a significance level of *p* ≤ 0.05. This test was chosen to control for Type I errors while making multiple comparisons between treatment groups. All results were expressed as mean ± standard deviation, and significance across treatments was evaluated based on the Tukey–Kramer test results.

## Data Availability

The datasets presented in this study can be found in online repositories. The names of the repository/repositories and accession number(s) can be found in the article/Supplementary material.
