# Supplementary figures and images for: Managing tomato bacterial wilt through pathogen suppression and host resistance augmentation using microbial peptide
Source: Front Microbiol. 2024 Dec 11;15:1494054. doi: 10.3389/fmicb.2024.1494054 (PMC11670319; doi:10.3389/fmicb.2024.1494054)

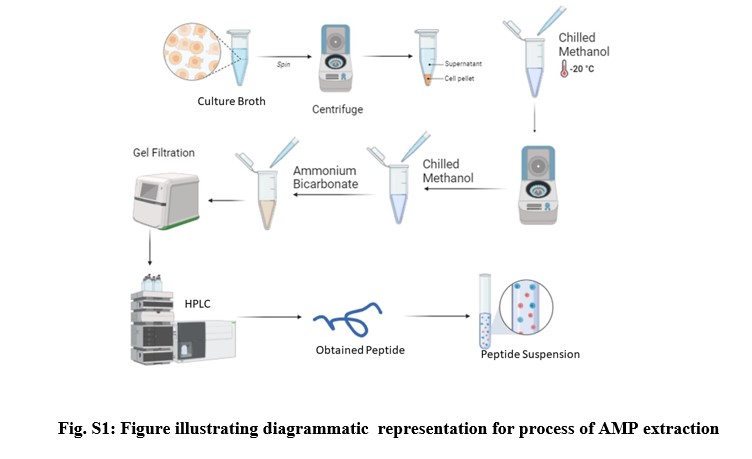

Supplement: Supplementary file 1 [file Image_1.jpeg]

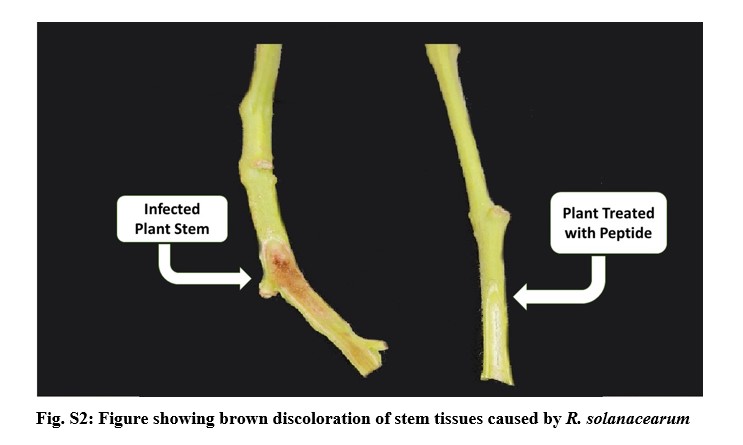

Supplement: Supplementary file 2 [file Image_2.jpeg]
